# Research on boundary optimization of adjacent mining areas in open pit coal mine based on calculation of sectional stripping ratio

**DOI:** 10.1038/s41598-023-48708-y

**Published:** 2023-12-02

**Authors:** Bo Cao, Jian Wang, Xiaobing Guo, Wenlu Li, Guangwei Liu

**Affiliations:** College of Mining, Liaoning Technical University, Fuxin, 123000 China

**Keywords:** Energy science and technology, Engineering

## Abstract

To address the problem of excessive local secondary stripping between adjacent mining areas in open pit mines caused by internal row raising, a multivariate function was fitted to the model of the main mining seam of Zhundong open pit coal mine in Xinjiang, and the different locations of the end gang of the second mining area were divided into multiple sections at certain step sizes and calculated by integration, resulting in stripping ratios for each section, which were fitted to a stripping ratio curve. The optimal location of the mining area boundary was found to be 55 m westward offset from the mining area boundary in the inner row of the raised section, and numerical simulations based on the strength reduction method were applied to analyse the slope stability of the end gang at this location. The results of the study show that the analysed slope meets the stability requirements and the optimised new boundary avoids the stripping of approximately 65,837,376 m^3^ of economically unreasonable section.

## Introduction

A large coal field is usually divided into a number of independent opencast mines, and the area divided into individual opencast mines is relatively large, and is subject to constraints in terms of equipment capacity, advancement intensity, capacity scale, initial and overall economic efficiency. The mine will be mined in a sequence from the first mining area until the entire mine is mined. Internal dump has significant economic and technical advantages over external dump^1–3^, such as less comprehensive transport work and a more orderly flow direction. If the next mining area is adjacent to the current mining area, and the current mining area has been filled with inward dump, the next mining area will often have to be stripped of some of the mine's dump material in order to extract as much of the coal resources as possible. Such “secondary stripping” increases the cost of stripping on the one hand, and on the other hand, more consideration should be given to safety, as the lower part of the end gang on the adjacent side of the two mining areas of the next mining area is overlaid with loose stripped material from the re-compacted internal dump field of the previous mining area, which generally has a low shear strength, so how to ensure the stability of the end gang on this side also requires The shear strength is generally low, so how to ensure the stability of the end gang on this side also requires specific study. “Secondary stripping” is often difficult to avoid, but the key to solving this problem is to optimise the mining area boundary to ensure slope stability while being economically optimal. In order to improve the economic efficiency of mining the overburdened coal seam in the overlapping part of the end gang of the adjacent open pit mine, Ma Li et al.^4^ derived a formula to calculate the effect of mining area width on the stripping ratio and constructed an optimisation model of mining area width and end gang coal compression height. Through theoretical analysis and calculation, Bai Runcai et al.^5^ derived the concept of common side gang pressure coal stripping ratio of adjacent inclined composite coal seam opencast mines and its calculation method, and then proposed a method to optimize the mining realm by eliminating the common side gang of adjacent inclined composite coal seam opencast mines. The method aims to release the internal dump space after mining the common side gang of adjacent opencast mines, increase the economic and reasonable stripping ratio of opencast mines, and improve the recovery rate of coal resources in mines. Bai Runcai et al.^6^ took West No. 2 Opencast Coal Mine and Ulantuga Opencast Coal Mine as the object of his research, formulated a coordinated mining plan for the two mines’ side gang pressed coal and optimized the mining area division plan, so that the side gang pressed coal of the two mines could be economically and reasonably backstepped, which improved the coal resource recovery rate and maximized the economic benefits. By analysing the factors influencing the economic stripping ratio of opencast mines, Liu Chuang et al.^7^ established a model for the location of stripping, mining and dump works in opencast mines with composite coal seams, constructed a fitting function for the thickness of the lower coal seam and its overburden, revealed the relationship between the mining realm of the lower coal seam and the mining cost, and researched and determined an optimal method for determining the economic stripping ratio of mining the lower coal seam. In this paper, through the investigation of the mining and dump status of many large-scale open-pit mines in China, it is found that a common type of soil dumping method is that the internal dump site continues to develop to a higher level beyond the original surface level after filling the mined pits. It is constructed in the way of “false outward soil discharge”. The internal row heightening and soil discharge scheme compensates to a certain extent the defects of the limited release of the inner row space and the long distance of the outer row, but in the long run, there will be more secondary stripping in the mining of adjacent mining areas in the future Quantity produced. Over the years, scholars have studied a series of theoretical calculation methods and boundary optimization schemes in order to maximize the mining of side-side compression coal seams, providing better solutions for similar problems faced by other open-pit mines.

**Figure 1:**
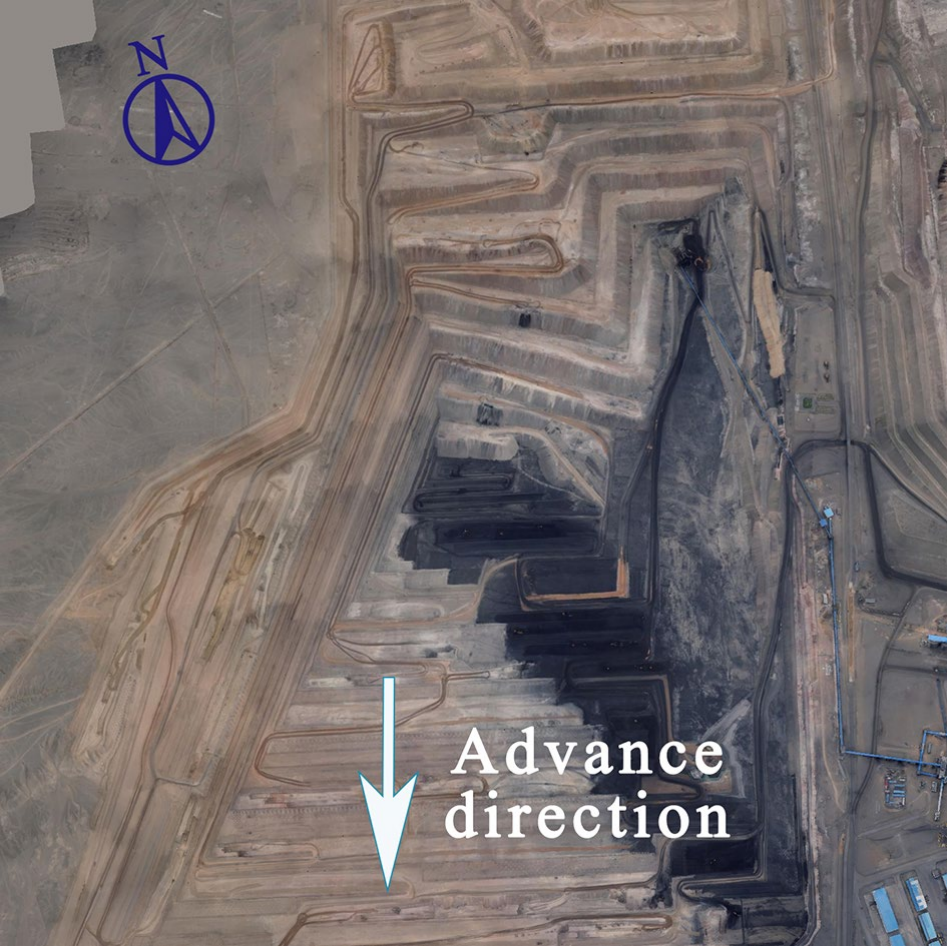
Current status of Zhundong Open-pit Coal Mine (The image is sourced from the author’s aerial photo).

However, little has been done on such issues. The Zhundong Open-pit Coal Mine studied in this paper is located in Jimsar County, Xinjiang Uygur Autonomous Region, China, and the current mining situation of this mine is shown in Fig. 1.

**Figure 2:**
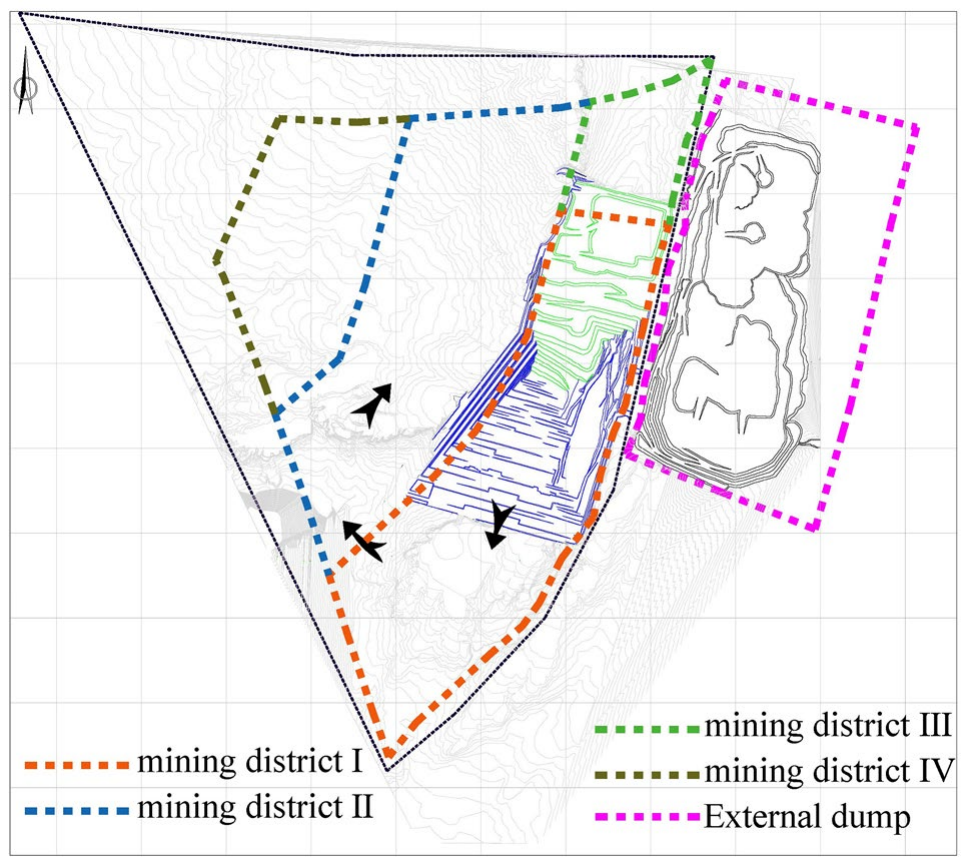
Schematic diagram of the division of mining areas in the Zhundong Open-pit Coal Mine.

The main coal seam of the Zhundong Open-pit Coal Mine is a huge inclined coal seam with a thickness of 70 m, and the tendency of the coal seam is to gradually decline from east to west. The whole mine is divided into four mining areas for mining. Currently, the mining stage of the first mining area is in progress, and the mining area is about to turn and transition to the second mining area. The division of mining areas is shown in Fig. 2. The inner row of the mine has been raised from level 576 to level 636, with a height of 60 m. Heightening the inner row certainly has obvious economic advantages.On the premise of retaining this rejection scheme, this paper constructs a more accurate coal seam reserve calculation model and a boundary optimization scheme, aiming at maximizing mining revenue under the premise of safety guarantee.

**Figure 3:**
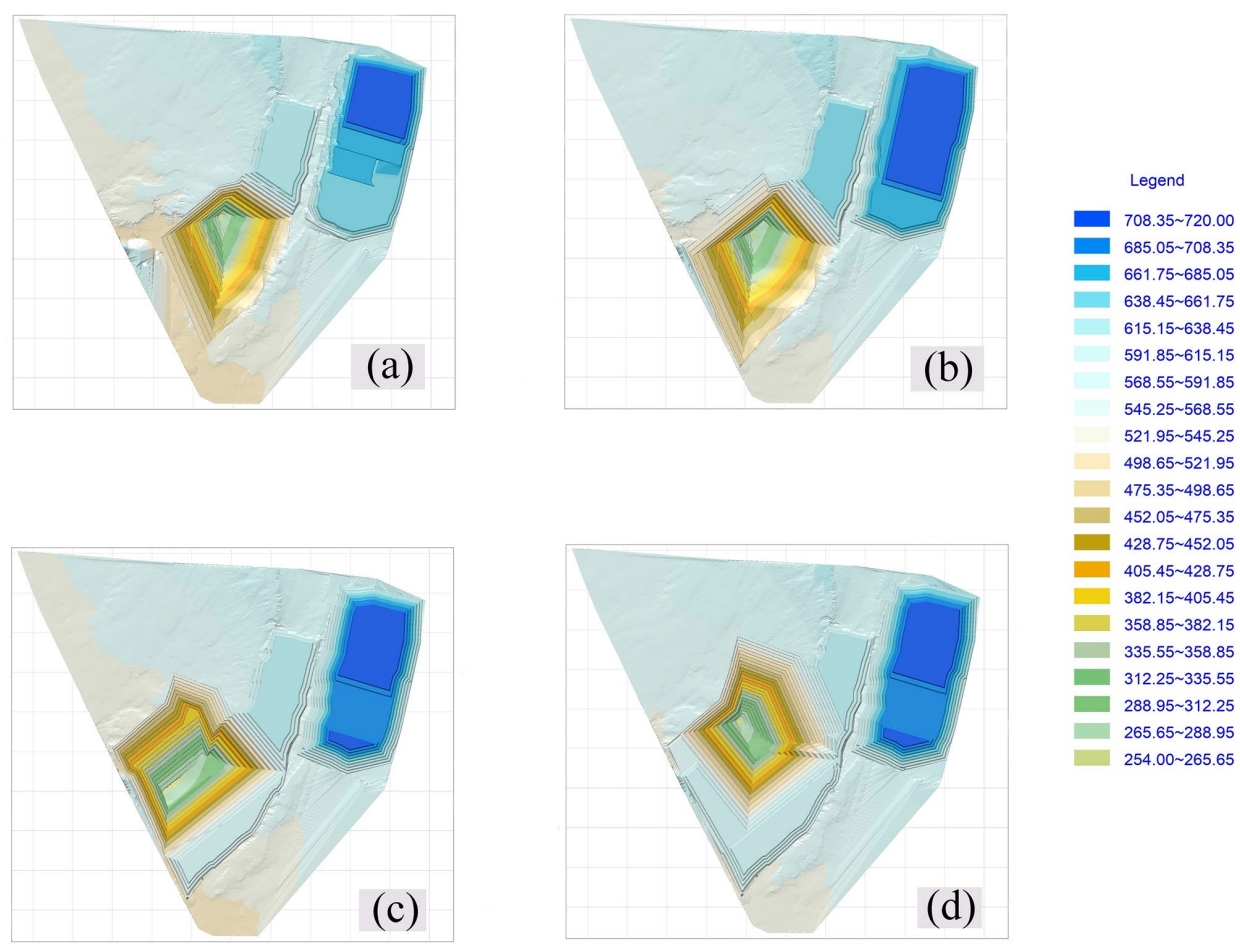
Simulated mining diagram: (**a**) position before turning; (**b**) starting turning position; (**c**) first 90° slow side turning completed when horizontal mining is transferred to vertical mining; (**d**) The second 90° slow side shift is completed and the second mining area is gradually mined from vertical mining to horizontal mining.

**Figure 4:**
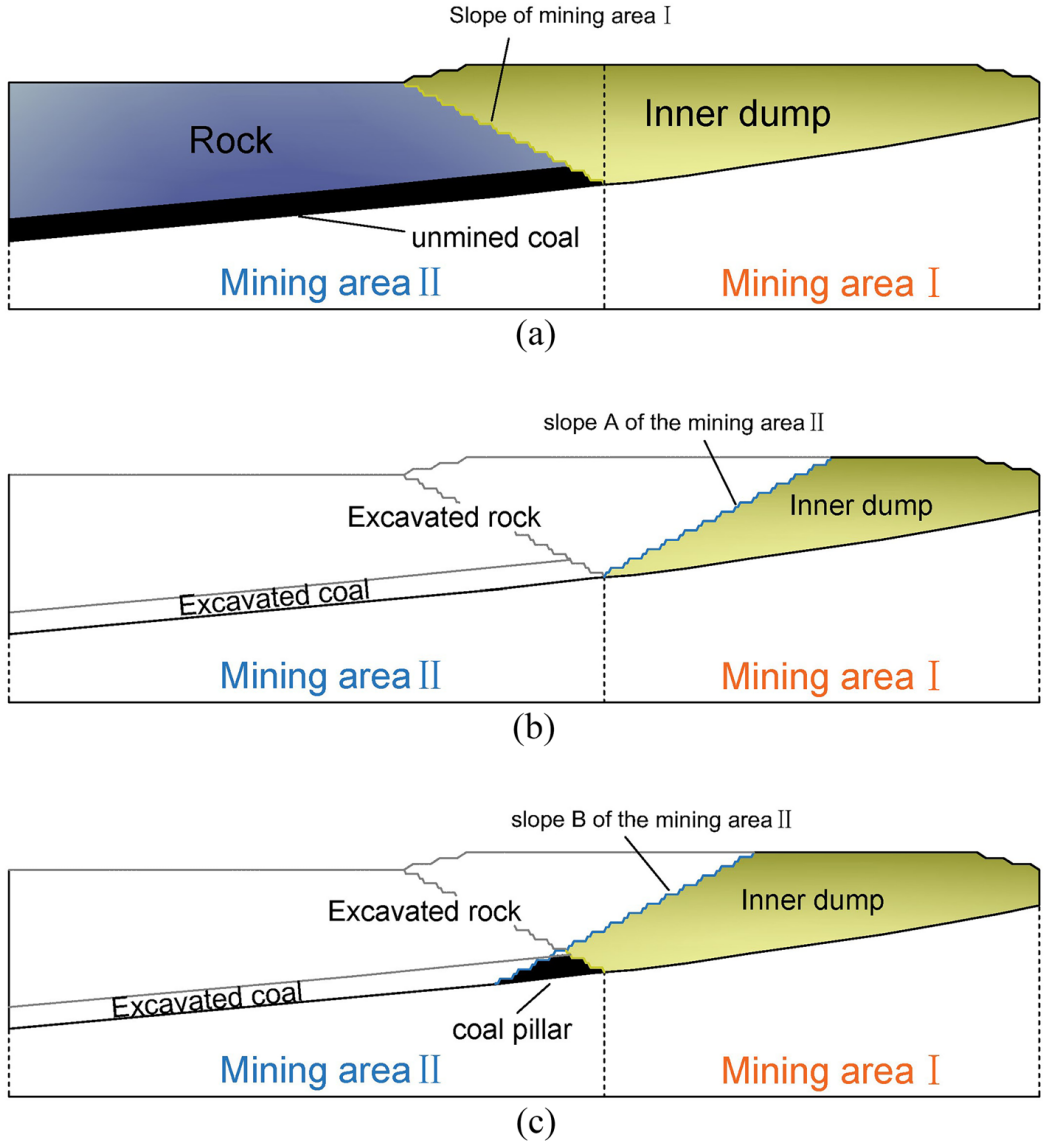
Schematic diagram of the edge position of the second mining area: (**a**) before the second mining area is mined; (**b**) all the main mining coal resources in the second mining area are mined; (**c**) some coal resources are reserved.

The mine has been simulated mining in the next few years, as shown in Fig. 3. The transition between the two mining intervals requires a 180° steering work, as can be seen from (d) in Fig. 3, If the coal resources existing in the second mining area are to be fully exploited, it is necessary to strip a large amount of compacted internal discharge materials in the first mining area, and the discharge height has exceeded 60 m above the ground surface. The secondary stripping here is relatively large.At the same time, it is difficult to ensure that the stripping ratio is within the economically reasonable threshold. Before exploring the boundary optimization method of the second mining area, two end-side boundary models were constructed, as shown in Fig. 4, in which (a) is the state before mining in the second mining area, and the lower coal seam covered by the end side of the first mining area is in a “ladder shape”, (b) is the boundary formed after mining all the coal seams, and (c) is the boundary of the mining area where part of the coal is sacrificed but the amount of stripping is less. In the following, the two mining boundaries will be used as the boundary conditions, and the optimum mining area boundary will be optimized by constructing a segmental stripping ratio calculation model and the best stable slope shape, and the optimal location of the second mining area to the boundary side will be explored.

## Methodology

### Multivariate function fitting of coal seam

**Figure 5:**
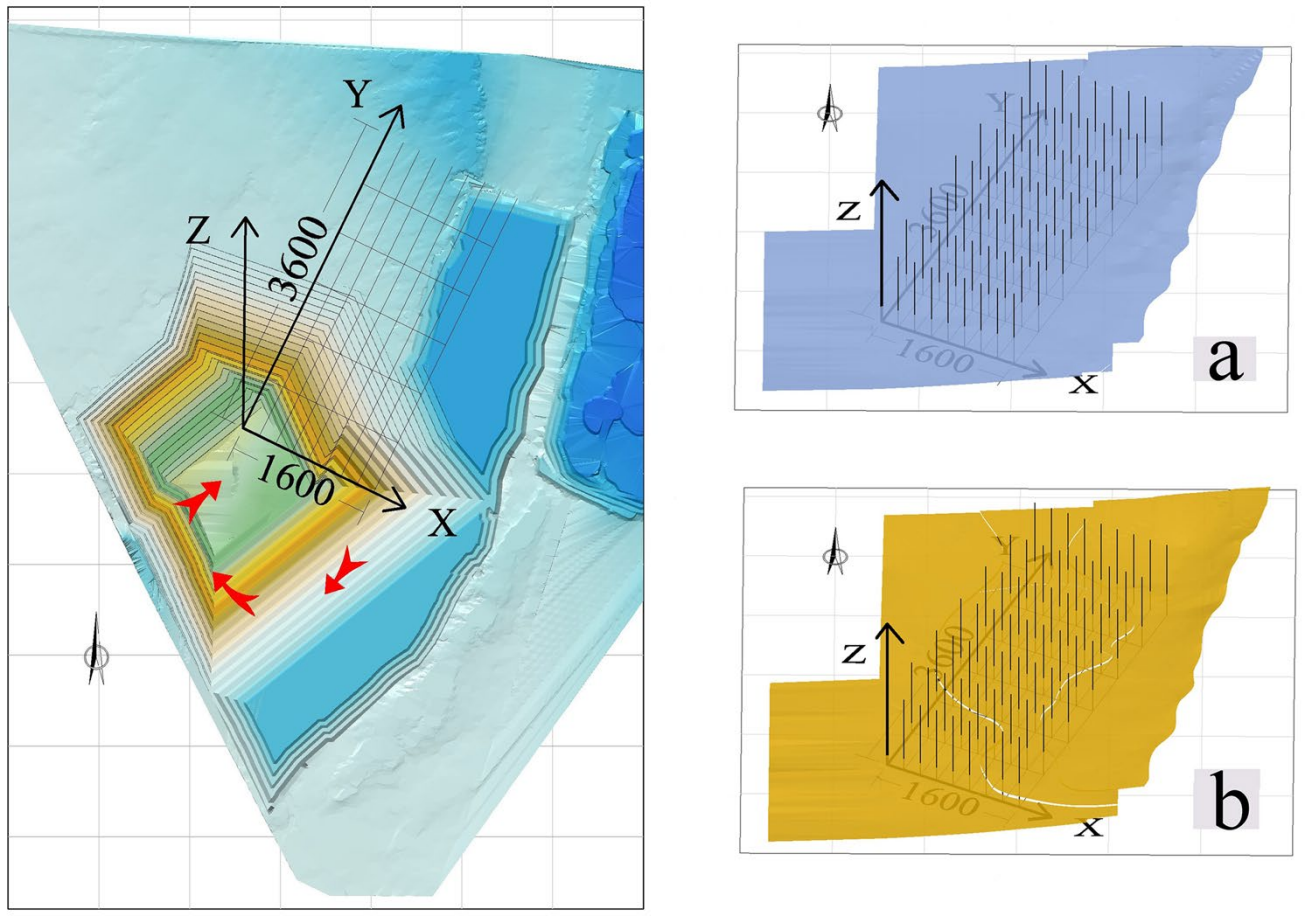
Construction of 3D coordinate system: (**a**) Construction of 3D coordinate system of coal seam roof and extraction of 63 elevation points; (**b**) Construction of 3D coordinate system of coal seam floor and extraction of 63 elevation points.

One of the keys to the analysis of the stripping ratio is the accuracy of the calculation of coal reserves. At present, software such as Datamine^8^, Vulcan^9^, and 3Dmine^10^ is used to calculate the coal reserves of open-pit mines, and the coal seam roof and floor models are constructed through drilling data. Then construct the block model, and use the area model to constrain the block model to estimate the reserves. However, the calculation accuracy of this method is greatly affected by the size of the block model. The smaller the block size in the model, the higher the accuracy, the larger the storage space required for the data, and the slower the computer running speed. Usually, the size of the block depends on the type, scale and mining method of the ore body. Each cuboid with a certain volume is superimposed to form a block model, and the block needs to be divided into smaller sub-blocks at the edge of the mine field. Make the block at the edge of the well field closer to the coal seam. Nevertheless, some errors still occur when constraining the margins, especially when dealing with coal seams with complex occurrences. Despite the existence of several resource estimation methodologies^11^, from traditional to geostatistical ones^12,13^ that use the block model as a geometrical representation, in the particular case of tabular or seam-shaped deposits these methods do not allow a sufficiently accurate representation or interpolation^14^. Advances have been made in the modeling of local anisotropy in complex geological structures, see^15^, but this method does not include mining parameters such as the minimum thickness valuesor the dilution. In view of the stable occurrence of coal seams in the Zhundong Open-pit Coal Mine, and also for those wanting results with smaller errors than those optimized by mining engineering software, this paper intends to use multiple integrals to estimate the stripping ratio. Prior to this, in order to calculate the reserves of the main coal seam, it is necessary to fit the roof and floor of the main coal seam to a multivariate function expression. As shown in Fig. 5, to ensure all data values are positive for ease of calculation, the first hexagram limit of the three-dimensional Cartesian coordinate system is constructed on the constructed roof and floor surface model of the main coal mining seam, and 63 three-dimensional coordinate points are extracted for the roof and floor respectively.

**Figure 6:**
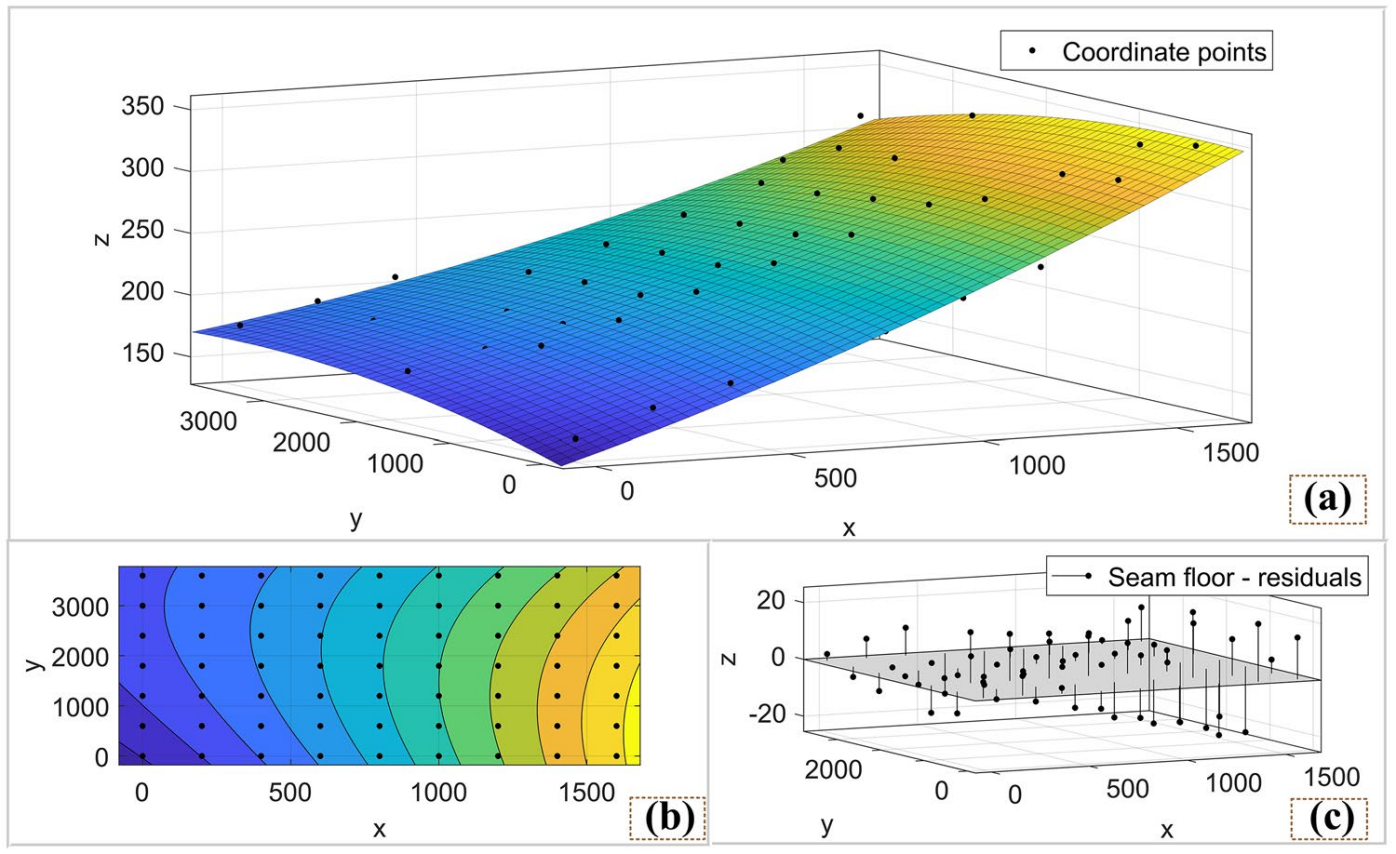
Coal seam floor surface fitted by matlab: (**a**) coal seam floor surface; (**b**) top view of contour line; (**c**) Three-dimensional coordinate point residual, it can reflect the degree of fit of the model.

**Figure 7:**
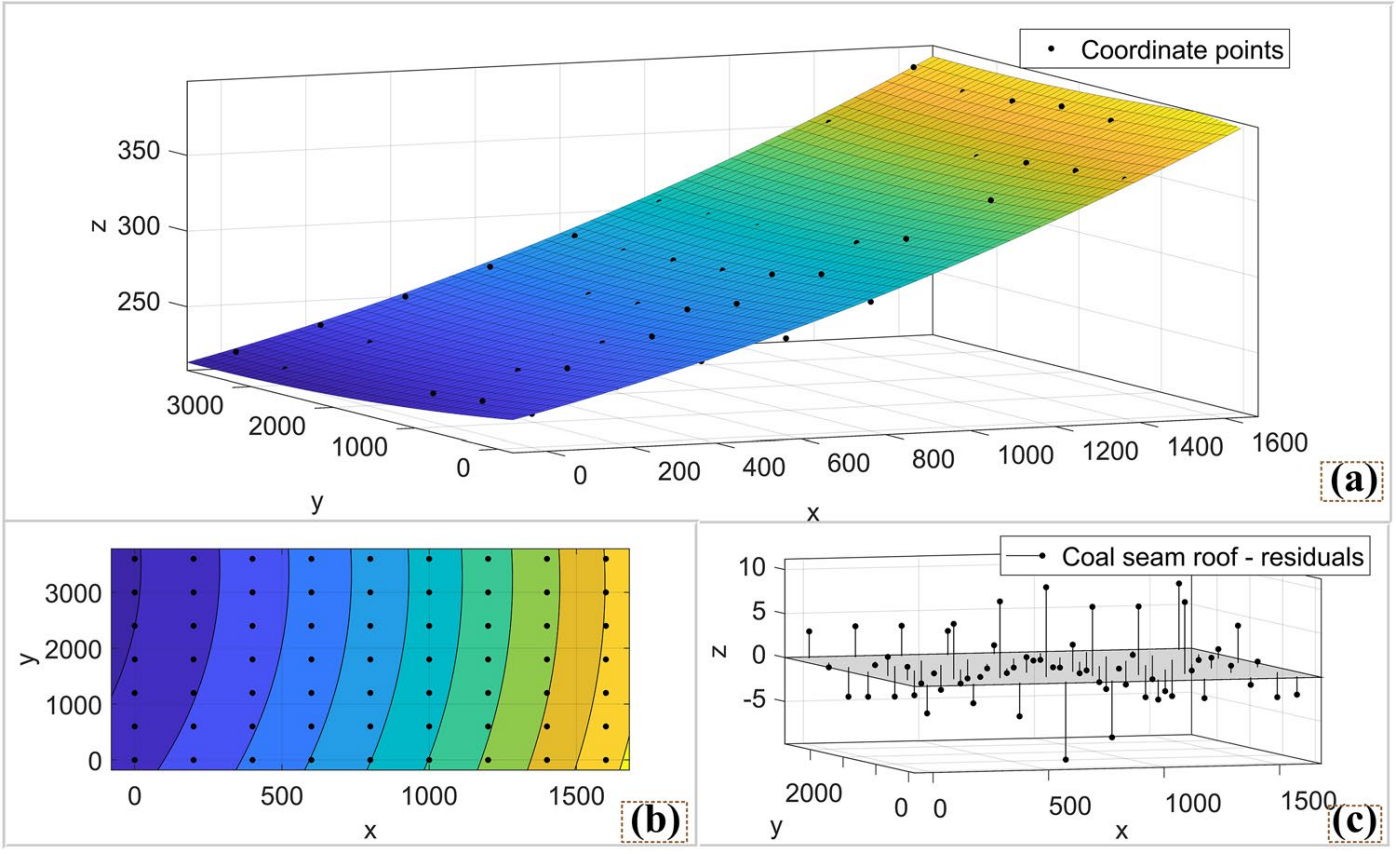
Coal seam roof surface fitted by matlab: (**a**) Coal seam roof surface; (**b**) Contour top view; (**c**) Three-dimensional coordinate point residual, it can reflect the degree of fit of the model.

Import the obtained 3D coordinate point data into Matlab software for polynomial fitting. The fitting results of the floor and roof are shown in Figs. 6 and 7. In order to ensure that the fitting results can truly reflect the occurrence of coal seams as much as possible, At the same time to avoid the phenomenon of over-fitting, the highest degree of fitting polynomial is selected twice. The fitted floor surface function is as formula, The roof surface function is as formula .

1 $$ Z_{f} = 141 + 0.09376x + 0.02236y + 0.00001749x^{2} - 0.00001181xy - 0.0000036y^{2} $$

2 $$ Z_{r} = 233.1 + 0.06675x - 0.008367y + 0.00002048x^{2} + 0.0000005xy + 0.000001196y^{2} $$

### Calculation of sectional stripping ratio based on numerical integral

**Figure 8:**
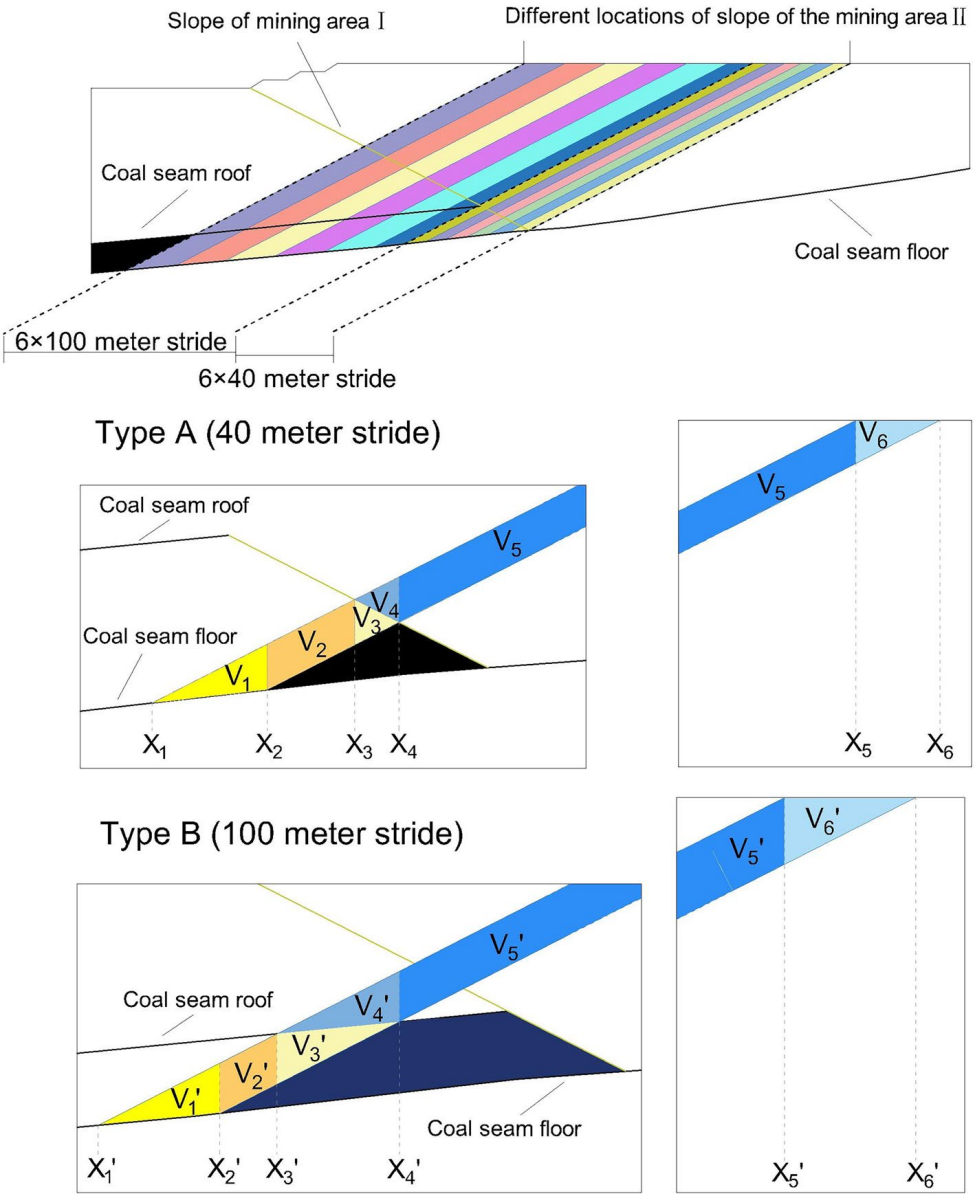
Selection of the position from the east side of the second mining area to the boundary side and the construction of the stripping ratio pre-calculation model. Due to the need for more accurate computational results in the boundary regions, the calculation zones are not divided with equal intervals. On the side closer to the boundary, six calculation zones with a step interval of 40 m are allocated, while on the distant side, six calculation zones with a step interval of 100 m are assigned.

In order to find the best position of the edge boundary on the side adjacent to the second mining area and the first mining area, a position model of the end edge of the boundary is constructed at a certain step distance, as shown in Fig. 8, a total of 12 end edge calculation sections are taken, according to the coal seam occurrence status, two types of section steps are divided. From the first mining area to the boundary side, the “triangular coal” is overlaid. Part of the coal resources here has been mined by the first mining area, and the stripping ratio may change greatly. As a key research area, therefore, six end-side calculation sections with a small step distance of 40 m are set here. In addition, six end-side calculation sections with a large step distance of 100 m are set up in the west to analyze the overall stripping ratio in a larger range.

As shown in Fig. 8, the coal seam in the 40 m step calculation section is not a complete coal seam compared with the 100 m step calculation section, so it is divided into Type-A and Type-B respectively to construct different numerical integral calculation models . As shown in Type-A in the Fig. 8, it represents any 40 m small section in the 6 calculation sections. In order to facilitate the calculation of multiple integrals, it is divided into multiple small volume calculation units according to the X coordinates of the intersection points of each curve. Before calculation, it is necessary to convert the various parameters of the open-pit mine mentioned above into a model that can meet the requirements of mathematical calculations. As in formulas and above, We have obtained the surface function expression by fitting the most complex coal seam roof and floor through three-dimensional coordinate points. Referring to the shape of the first mining area to the boundary end, it is proposed to express the boundary side of different positions in the second mining area as an inclined plane at 28° to the horizontal plane. According to the three-dimensional rectangular coordinate system constructed above, The function expression of the rightmost side of the 40 m step distance calculation section is as follows:

3 $$ {\text{Z}}_{B1} { = 0}{\text{.5435x}} - {583}{\text{.6}} $$

The function expression of the boundary side 40 m away from its west is as follows:

4 $$ {\text{Z}}_{B2} { = 0}{\text{.5435x}} - {561}{\text{.9}} $$

**Figure 9:**
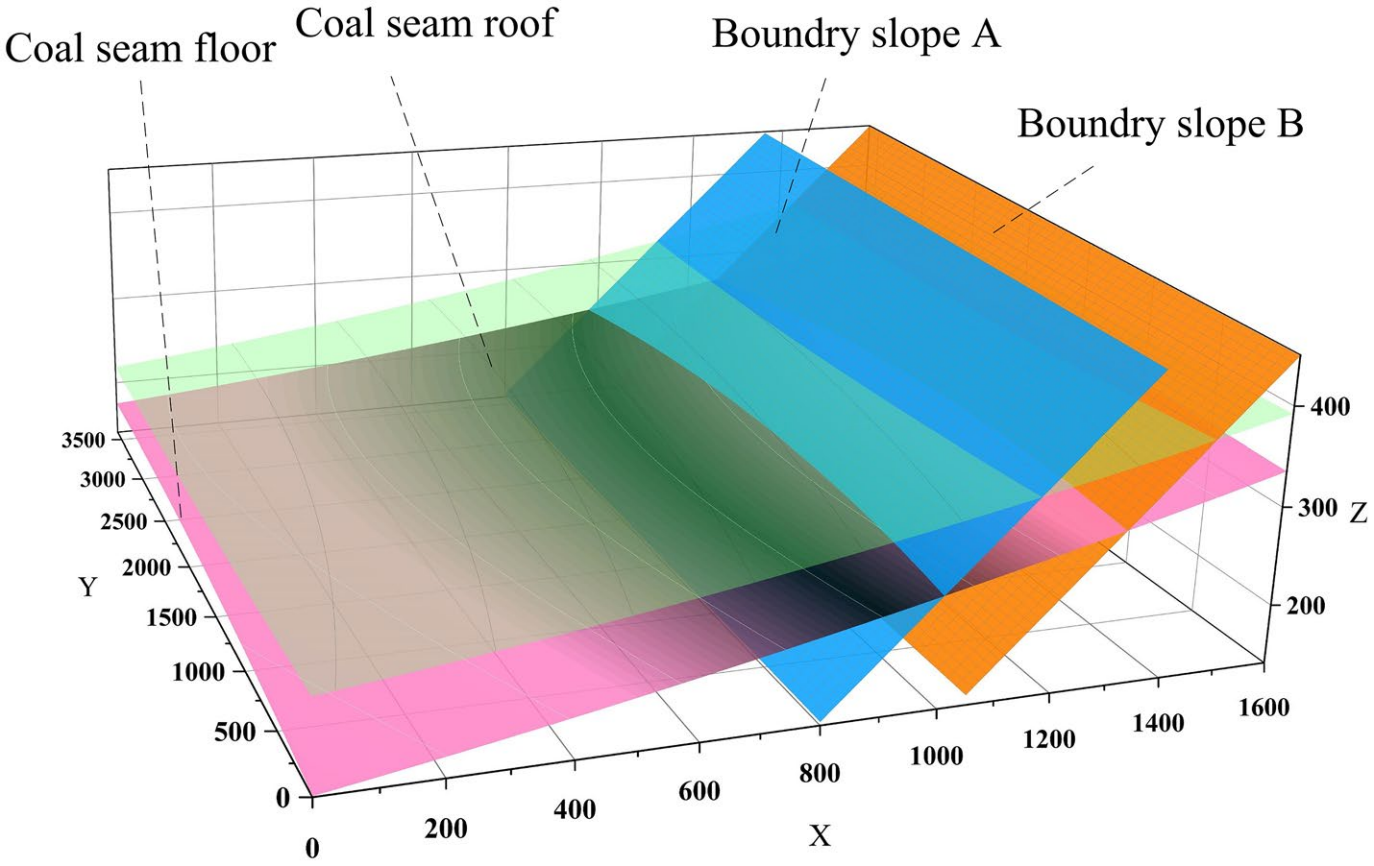
The relationship between the end side position and the curved surface of the roof and floor of the coal seam.

The function expressions of the other 11 end-side positions are constructed in the same way, and will not be repeated here. The positional relationship of the two end slopes and the roof and floor surfaces of the coal seam in the first hexagram limit of the three-dimensional coordinate system is shown in Fig. 9.

The X coordinates in Type-A are obtained by combining the above two intersecting surface functions. After the combination, the result is generally a curve function. The calculation process and results are relatively simple, and will not be described here. The obtained results are directly used below. Taking the second calculation section in the middle and eastern part of Type-A as an example, the numerical integration for each section was carried out using the software Wolfram Mathematica. The process and results are detailed below:

5 $$ \begin{aligned} V_{1} = & \mathop \smallint \nolimits_{0}^{3600} 1\mathop \smallint \nolimits_{{\frac{{44974000 + 1181y - \sqrt {1546163116000000 + 90585532000y + 3913321y^{2} } }}{3498}}}^{{\frac{{44974000 + 1181y - \sqrt {1530911836000000 + 90585532000y + 3913321y^{2} } }}{3498}}} ( - 681.1 + 0.44974x \\ & - 0.00001749x^{2} - 0.02236y + 0.00001181xy + 3.6 \times 10^{ - 6} y^{2} )dxdy \\ \end{aligned} $$

6 $$ V_{2} = \mathop \smallint \nolimits_{0}^{3600} 1\mathop \smallint \nolimits_{{\frac{{44974000 + 1181y - \sqrt {1530911836000000 + 90585532000y + 3913321y^{2} } }}{3498}}}^{1680} (21.8)dxdy $$

7 $$ V_{3} = \mathop \smallint \nolimits_{0}^{3600} 1\mathop \smallint \nolimits_{1680}^{1700} \left[ { - 0.5435x + 1285 - (0.5435x - 561.9)} \right]dxdy $$

8 $$ V_{4} = \mathop \smallint \nolimits_{0}^{3600} 1\mathop \smallint \nolimits_{1680}^{1700} \left[ {0.5435x - 540.1 - ( - 0.5435x + 1285)} \right]dxdy $$

9 $$ V_{5} = \mathop \smallint \nolimits_{0}^{3600} 1\mathop \smallint \nolimits_{1700}^{2164} \left[ {0.5435x - 540.1 - (0.5435x - 561.9)} \right]dxdy $$

10 $$ V_{6} = \mathop \smallint \nolimits_{0}^{3600} 1\mathop \smallint \nolimits_{2164}^{2204} \left[ {636 - (0.5435x - 561.9)} \right]dxdy $$

In the above,${V}_{1}$, ${V}_{2}$, ${V}_{3}$ is the partial volume of the coal seam in the side calculation section ${V}_{4}$, ${V}_{5}$, ${V}_{6}$ is the volume of rock and soil. The bulk density of coal γ is 1.29 t/m^3^, total volume of coal is ${V}_{c}$, total volume of rock is ${V}_{r}$, Then the stripping ratio ${n}_{q}$ is:

11 $$ n_{q} = \frac{{V_{r} }}{{V_{c} \times \gamma }} = \frac{{V_{3} + V_{4} + V_{5} }}{{\left( {V_{1} + V_{2} + V_{3} } \right) \times \gamma }} $$

Substitute formula ~ into formula The stripping ratio ${n}_{q}$ between calculation sections can be obtained, The calculation process of the other five calculation sections is basically similar, and will not be repeated here. For Type-B, the coal seam between each calculation section consisting of two end-to-end positions is complete, therefore, in addition to calculating and summing each sub-volume as in Type-A, this paper takes the westernmost calculation section of Type-B as an example to propose another calculation method, the distinctive feature of this computational method is the amalgamation of volume sections V_2_ to V_5_ into a single larger section for calculation. After multiple validations, it has been demonstrated that this approach results in reduced errors.

12 $$ \begin{aligned} V_{1}^{\prime } = & \mathop \smallint \nolimits_{0}^{3600} 1\mathop \smallint \nolimits_{{\frac{{44974000 + 1181y - \sqrt {1816488556000000 + 90585532000y + 3913321y^{2} } }}{3498}}}^{{\frac{{44974000 + 1181y - \sqrt {1781893336000000 + 90585532000y + 3913321y^{2} } }}{3498}}} ( - 294.7 + 0.44974x \\ & - \left. {0.00001749x^{2} - 0.02236y + 0.00001181xy + 3.6 \times 10^{ - 6} y^{2} } \right)dxdy \\ \end{aligned} $$

13 $$ \begin{gathered} V^{\prime}_{2\sim 5} = V^{\prime}_{2} + V^{\prime}_{3} + V^{\prime}_{4} + V^{\prime}_{5} = \mathop \smallint \nolimits_{0}^{3600} 1\mathop \smallint \nolimits_{{\frac{{44974000 + 1181y - \sqrt {1781893336000000 + 90585532000y + 3913321y^{2} } }}{3498}}}^{1453} \\ 0.5435x - 153.7 - (0.5435x - 203.15)dxdy \\ \end{gathered} $$

14 $$ V^{\prime}_{6} = \mathop \smallint \nolimits_{0}^{3600} 1\mathop \smallint \nolimits_{1453}^{1544} 636 - ( - 203.15 + 0.5435x)dxdy $$

15 $$ \begin{aligned} V_{4}^{\prime } = & \mathop \smallint \nolimits_{0}^{3600} 1\mathop \smallint \nolimits_{{\frac{{119187500 - 125y - \sqrt 5 \sqrt {2445048831250000 + 2608433000y - 1221579y^{2} } }}{10240}}}^{{\frac{{119187500 - 125y - \sqrt 5 \sqrt {2394412031250000 + 2608433000y - 1221579y^{2} } }}{10240}}} ( - 386.8 + 0.47675x \\ & - \left. {0.00002048x^{2} + 0.008367y - 5 \times 10^{ - 7} xy - 1.196 \times 10^{ - 6} y^{2} } \right)dxdy \\ \end{aligned} $$

16 $$ \begin{aligned} V_{5}^{\prime } = & \mathop \smallint \nolimits_{0}^{3600} 1\mathop \smallint \nolimits_{{\frac{{119187500 - 125y - \sqrt 5 \sqrt {2394412031250000 + 2608433000y - 1221579y^{2} } }}{10240}}}^{1453} 0.5435x \\ & - 153.7 - (0.5435x - 203.15)dxdy \\ \end{aligned} $$

Therefore, the calculation of the stripping ratio $n^{\prime}_{q}$ is as follows:

17 $$ n^{\prime}_{q} = \frac{{V^{\prime}_{4} + V^{\prime}_{5} + V^{\prime}_{6} }}{{(V^{\prime}_{1} + V^{\prime}_{2\sim 5} - V^{\prime}_{4} - V^{\prime}_{5} ) \times \gamma }} $$

So far, the calculation methods of the stripping ratio based on numerical integration for Type-A and Type-B, which are composed of 13 boundary positions and a total of 12 calculation sections, have been given.

## Results

### Determination of the boundary position of the mining area II

Through the above method, the total amount of coal and rock and the stripping ratio of all sections can be calculated, and the abscissa of the intersection of each boundary end and the surface is used as the boundary. The overall calculation results are shown in the Table 1.

**Table 1:** Calculation results of stripping ratio in each section.

Classification	Calculation section	Volume of rock (m^3^)	Coal weight (t)	Sectional stripping ratio (m^3^/t)	Cumulative stripping ratio (m^3^/t)
Type-B	1453–1544	110,600,000	28,921,800	3.82	4.05
	1544–1644	116,980,000	32,121,000	3.64	3.99
	1644–1744	75,900,000	20,046,600	3.79	3.98
	1744–1844	150,680,000	43,942,000	3.43	3.90
	1844–1944	101,080,000	33,798,000	2.99	3.81
	1944–2004	57,840,000	20,253,000	2.86	3.76
Type-A	2004–2044	32,480,400	19,903,668	1.63	3.65
	2044–2084	33,990,400	16,936,668	2.01	3.58
	2084–2124	35,730,400	13,556,868	2.64	3.55
	2124–2164	37,106,080	10,250,753	3.62	3.55
	2164–2204	38,828,960	7,015,846	5.53	3.58
	2204–2244	42,540,000	2,639,985	16.11	3.66

Combining the coal seam occurrence characteristics and mining status of the open-pit mine, the above data can be concluded as follows: Since the coal seam tends to be from west to east, for the second mining area, the closer the coal seam is to the junction of the first mining area and the second mining area, the shallower the coal seam is buried, and the less rock needs to be stripped for coal mining, the stripping ratio may gradually increase according to the deepening of the coal seam burial. However, the thickness of the coal seam is not uniform, and it will also have a considerable impact on the change of the stripping ratio. It can also be seen from the calculation results in the table that in the calculation section Type-B, the section stripping ratio generally decreases gradually with the decrease of the coal seam burial depth. As in Fig. 8 above, Type-A is mainly a triangular incomplete coal seam, and the volume of the coal seam gradually decreases from west to east (the positive direction of the X-axis of the construction coordinate), so the section stripping ratio of Type A in the above table gradually increases, the model corresponds exactly to the data results. The three sections of 1944–2004, 2004–2044, and 2044–2084 are the transition areas where the stripping ratio of the section decreases from decreasing to increasing, and this area is also the junction area between Type-B and Type-A. The reason for this change is that the burial depth of the coal seam gradually decreases, and the occurrence of the coal seam gradually transitions from the complete coal seam to the triangular residual coal.

**Figure 10:**
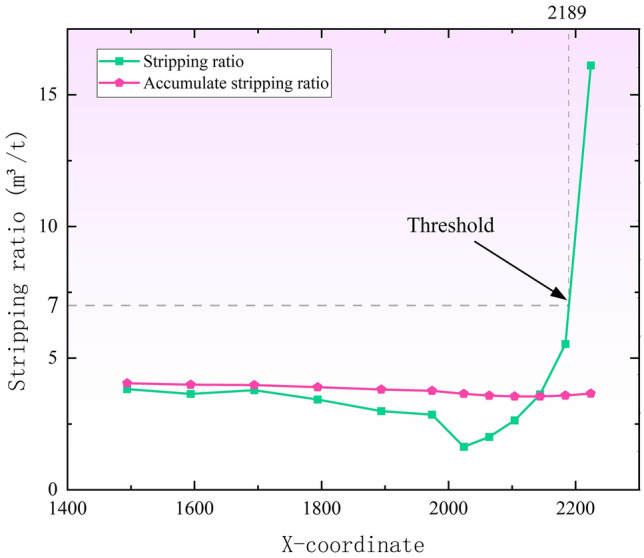
Stripping ratio curve.

**Figure 11:**
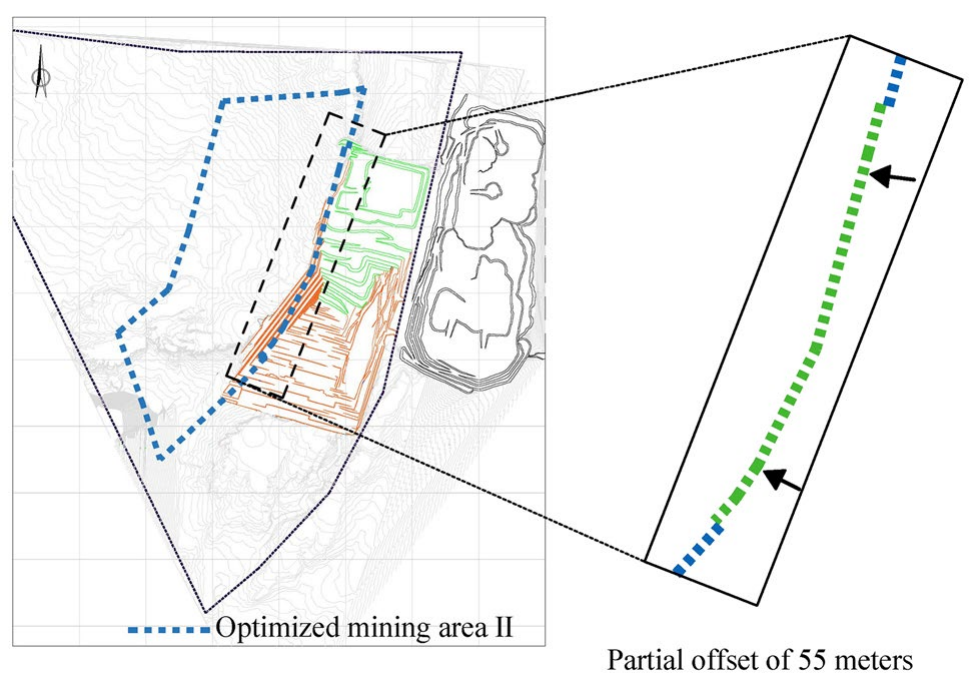
Optimized deep boundary of the second mining area.

The boundary stripping ratio of open-pit mining generally has a threshold, which is an economic stripping ratio calculated comprehensively from various mining costs and coal selling prices^16^. The economic stripping ratio of this mine is 7. Based on the economic stripping ratio to locate a reasonable boundary position, this paper conducts linear fitting analysis on the data in the table, the line graph is shown in Fig. 10, and the cumulative stripping ratio is used as a reference index, it is distinguished from the stripping ratio calculated by sections, instead, it represents a cumulative calculation of the total stripping and coal extraction across the entire mining area, and its value does not change much; The section stripping ratio showed a trend of decreasing and then rising, and the rising speed gradually increased. When the economic stripping ratio was 7, its X coordinate is 2189, and X = 2189 is the abscissa of the intersection point from the second mining area to the boundary end and the surface in the three-dimensional coordinate system. The original design was X = 2244, from this, it can be determined that the best boundary position of the end side of the second mining area is the difference distance between the original design plan and the westward offset between the two, that is, 55 m. Therefore, the boundary of the second mining area is re-divided as shown in Fig. 11. Compared with the original boundary, the optimized boundary has reduced the stripping volume of the relatively large area by about 65,837,376 m^3^.

### Stability analysis of the east side slope in the mining area II after optimization

**Figure 12:**
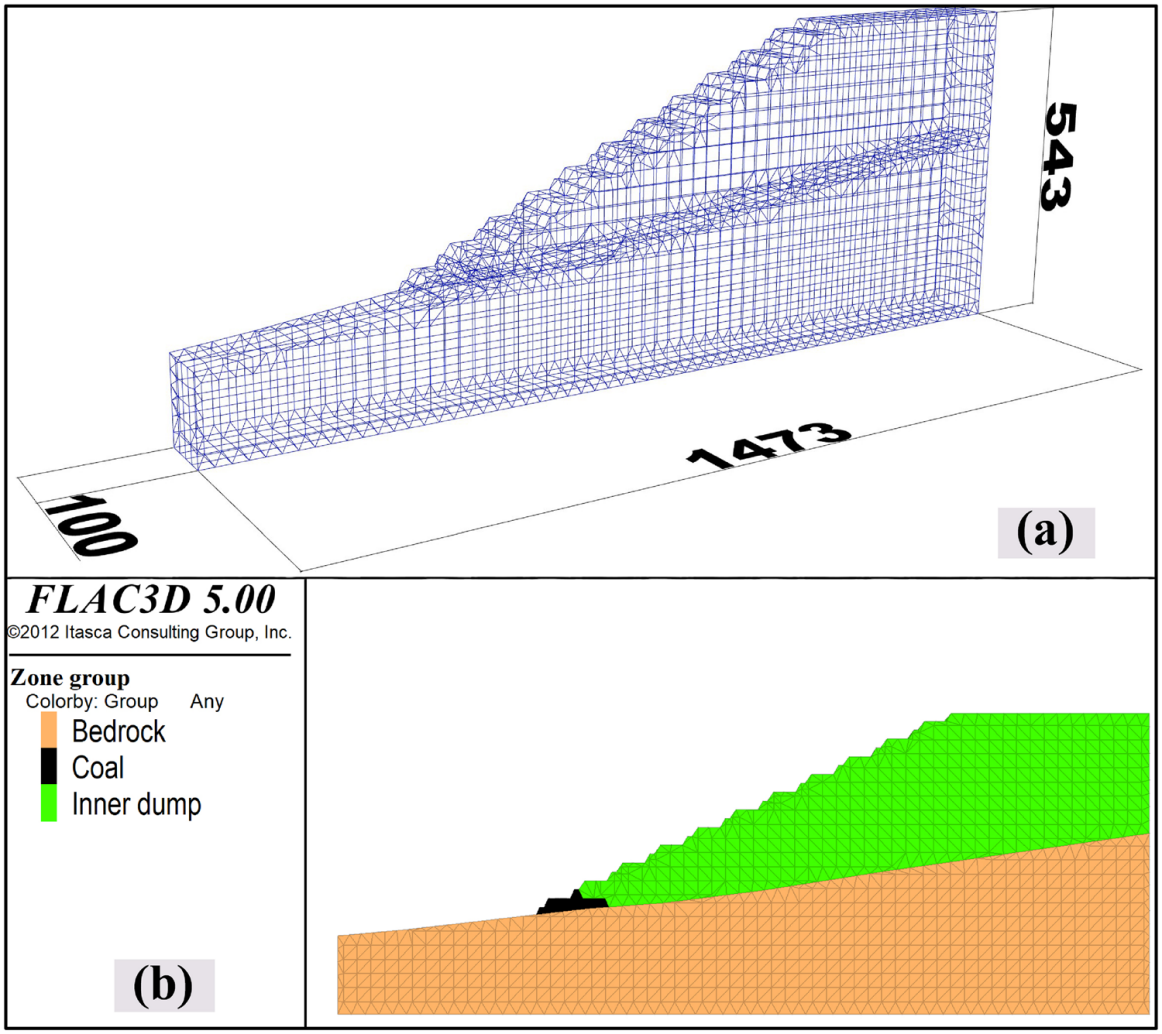
Slope 3D numerical model construction: (**a**) 3D grid model established by Rhino software; (**b**) slope model grouping.

In the above, the boundary optimization plan of the second mining area has been obtained through numerical calculation and stripping ratio section analysis, and the relatively large stripping part is divided out of the boundary to reduce local losses and improve economic benefits, however, ensuring the safety and stability of the slope is a necessary prerequisite for open-pit mining. In the calculation above, the preset end-side angle is the same as that of the first mining area, which is 28°. The west of the second mining area to the lower part of the boundary end side is the internal discharge of materials in the first mining area, and its cohesion is low. Therefore, it is planned to use the Flac3D numerical simulation based on the strength reduction method to analyze the slope stability^17–20^. The preparation for numerical simulation is the construction of the slope model, as shown in Fig. 12a, it is to construct a three-dimensional mesh model through Rhino software, and the size of the model is marked in the Fig. 12b, it is the grouping of the 3D model according to the coal-rock stratification.

**Figure 13:**
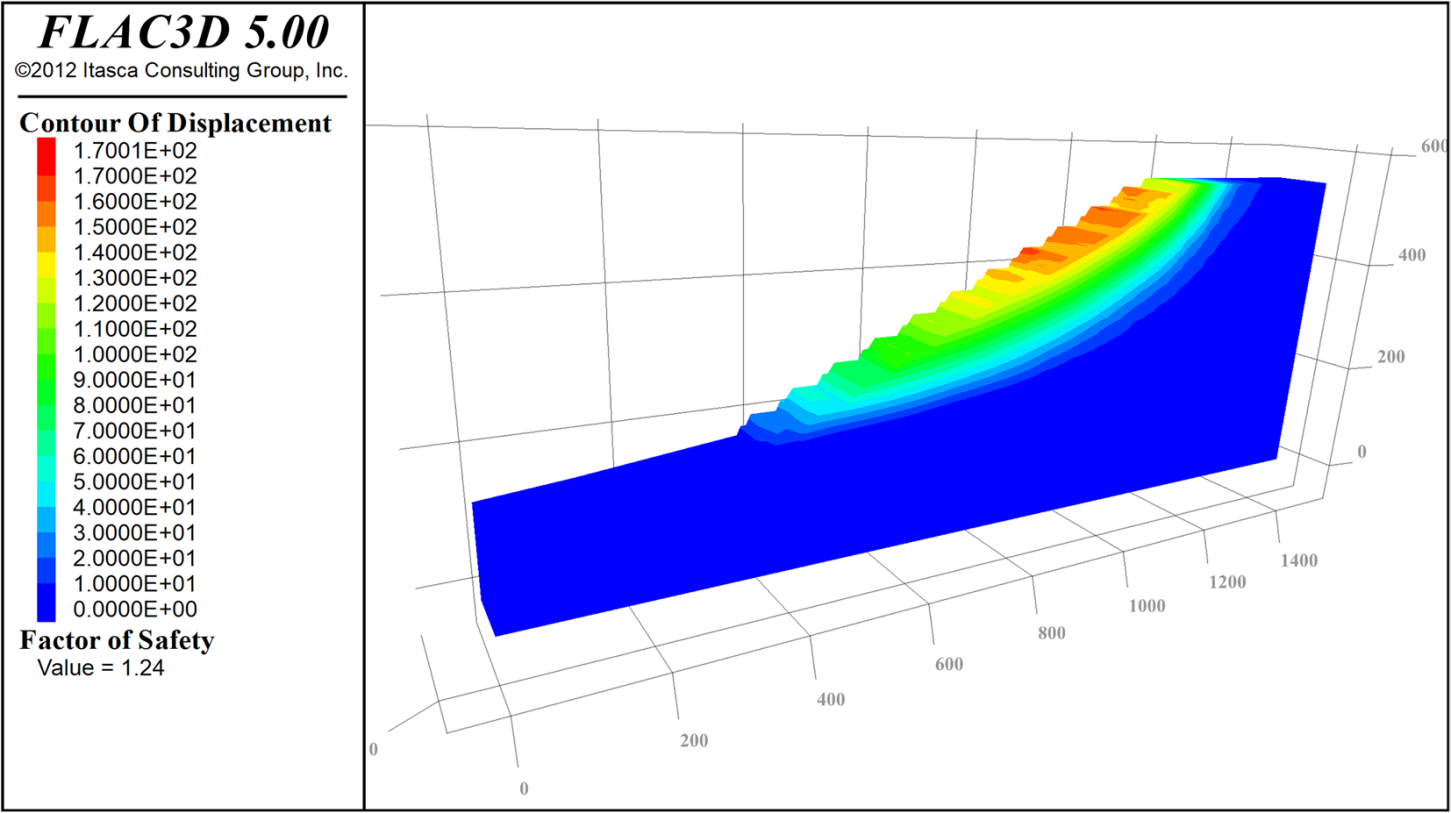
Calculated cloud map of slope displacement.

**Figure 14:**
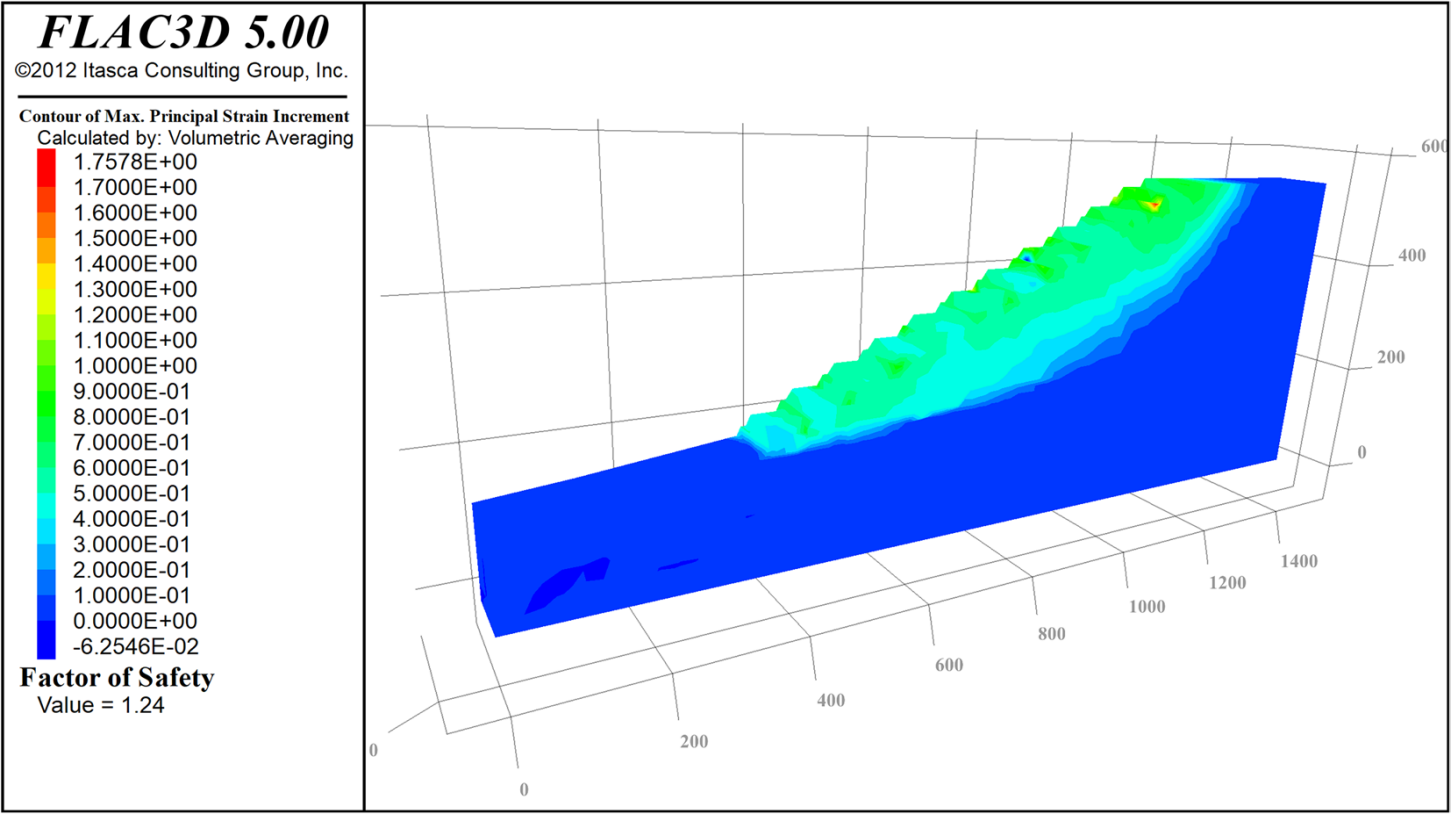
Cloud chart of maximum principal strain increment.

Based on the above three-dimensional model for slope stability calculation, based on the previous experimental data of the open-pit mine, the main mechanical parameters of each group are as follows: the cohesion of Bedrock is 0.136 Mpa, and the internal friction angle is 36°; the cohesion of Coal is 0.06 Mpa, the internal friction angle is 28°; the cohesive force of the inner dump is 0.012 Mpa, and the internal friction angle is 24°. The normal displacement constraints are carried out on the surrounding and bottom surfaces of the 3D model of the slope, and the top surface is the free end, and the slope stability is calculated using the strength reduction method. Figure 13 is a cloud map of the slope displacement. Figure 14 is the cloud diagram of the maximum principal strain increment. The calculated safety factor is 1.24, which is greater than the safety reserve factor of 1.2, so the slope is considered safe and stable.

## Conclusions

By analyzing the influence of the heightening of rows in the first mining area of Xinjiang Zhundong Open-pit Coal Mine on the stripping ratio in the mining process of the second mining area, in the second mining area, multiple calculation sections are established at a certain step distance from the end side position where all coal seams can be mined on the westernmost side, and the types of calculation sections are divided according to the remaining state of the coal seam, the triangular coal area is defined as Type-A, and the complete coal area is defined as Type-B, and the volume of each part is calculated by the numerical integration method, and the stripping ratio of each part of rock and coal is summarized. According to the constraints of the stripping ratio curve and the economically reasonable threshold value of the stripping ratio, the optimal side profile position is found, and the slope stability is calculated by numerical simulation method. The determination of this boundary optimization scheme has reduced the stripping volume in the area with a relatively large stripping ratio by about 65,837,376 m^3^, concurrently, there has been a reduction in the coal mining volume by an estimated 5,270,927 m^3^. Extracting this coal would lead to a financial deficit. Thus, foregoing its extraction emerges as a more economically prudent decision. In addition to the Zhundong Open-pit Coal Mine involved in this research, there are also many open-pit coal mines that are experiencing or will face similar difficulties in the future. Scholars have seldom studied this. This paper gives specific calculation methods and research schemes. It not only makes a boundary optimization scheme for the Zhundong Open-pit Coal Mine, but also provides research ideas for other open-pit coal mines. This article also has certain deficiencies. In terms of coal seam surface function fitting, the maximum order of the function independent variable is quadratic. Although the function fitting with a higher order may have a certain degree of overfitting, it also reduces the error to a certain extent. Higher-order functions will bring great difficulty to the integral calculation. This paper has made many trade-offs in the selection of the fitting function, and finally determined it as the quadratic surface function in this paper.

Today, when open-pit coal mines tend to be large-scale, the impact of a seemingly insignificant boundary change of tens of meters or even a few meters on economic benefits will be considerable. This paper studies the influence of the stripping ratio of adjacent mining intervals in open-pit mines that have been subjected to internal dump and heightening, and gives a boundary optimization scheme. For open-pit mines that have not yet decided whether to carry out internal dump and heightening, the long-term impact should be considered. If there is a secondary stripping, the overall benefit can be calculated according to the method given in this article, and then the final decision can be made.

## Data Availability

The data used to support the findings of this study are available from the corresponding author upon request.
